# Nitrogen fertilizer regulates soil respiration by altering the organic carbon storage in root and topsoil in alpine meadow of the north-eastern Qinghai-Tibet Plateau

**DOI:** 10.1038/s41598-019-50142-y

**Published:** 2019-09-24

**Authors:** Wen Li, Jinlan Wang, Xiaolong Li, Shilin Wang, Wenhui Liu, Shangli Shi, Wenxia Cao

**Affiliations:** 1 Grassland Ecosystem Key Laboratory of Ministry of Education, Sino-U.S. Research Centers for Sustainable Grassland and Livestock Management, Grassland Science College of Gansu Agricultural University, Lanzhou, 730070 People’s Republic of China; 2 Grassland Institute, Qinghai Academy of Animal Science and Veterinary Medicine, Xining, 810003 People’s Republic of China

**Keywords:** Ecology, Grassland ecology

## Abstract

Soil respiration (Rs) plays a critical role in the global carbon (C) balance, especially in the context of globally increasing nitrogen (N) deposition. However, how N-addition influences C cycle remains unclear. Here, we applied seven levels of N application (0 (N0), 54 (N1), 90 (N2), 126 (N3), 144 (N4), 180 (N5) and 216 kg N ha^−1^ yr^−1^ (N6)) to quantify their impacts on Rs and its components (autotrophic respiration (Ra) and heterotrophic respiration (Rh)) and C and N storage in vegetation and soil in alpine meadow on the northeast margin of the Qinghai-Tibetan Plateau. We used a structural equation model (SEM) to explore the relative contributions of C and N storage, soil temperature and soil moisture and their direct and indirect pathways in regulating soil respiration. Our results revealed that the Rs, Ra and Rh, C and N storage in plant, root and soil (0–10 cm and 10–20 cm) all showed initial increases and then tended to decrease at the threshold level of 180 kg N ha^−1^ yr^−1^. The SEM results indicated that soil temperature had a greater impact on Rs than did volumetric soil moisture. Moreover, SEM also showed that C storage (in root, 0–10 and 10–20 cm soil layers) was the most important factor driving Rs. Furthermore, multiple linear regression model showed that the combined root C storage, 0–10 cm and 10–20 cm soil layer C storage explained 97.4–97.6% variations in Rs; explained 94.5–96% variations in Ra; and explained 96.3–98.1% in Rh. Therefore, the growing season soil respiration and its components can be well predicted by the organic C storage in root and topsoil in alpine meadow of the north-eastern Qinghai-Tibetan Plateau. Our study reveals the importance of topsoil and root C storage in driving growing season Rs in alpine meadow on the northeast margin of Qinghai-Tibetan Plateau.

## Introduction

Human activities such as agricultural expansion, industrial development and deforestation have enormously altered the rate of N deposition^1,2^. Terrestrial ecosystems have been experiencing approximately twice the input of N worldwide compared to the level of the pre-industrial era^2^. According to the results of Lamarque *et al*.^3^, N deposition in the global terrestrial ecosystems will range from 60 to 100 Tg (N) per year by 2100. Moreover, the rate of N deposition will increase 2.5-fold in the next century^4^. Excess N has caused a range of ecological problems such as a reduction of biological diversity, affecting net primary productivity, community structure and alterations to the C cycle^5,6^. One of the main scientific problems to be addressed is to determine how N deposition influences C cycle^7,8^. Grasslands serve as a very important C pool. They store approximately 34% of the terrestrial global C stock^9^. Soil respiration (Rs) is an important source of C efflux from soil surface to atmosphere^10^. A small change in Rs can alter the concentration of CO_2_ in the atmosphere^11,12^. Therefore, understanding the magnitude of CO_2_ fluxes emitted from grassland and grassland C pool are crucial to estimate the contribution of grassland ecosystems to the global C budget^13^. Although there have been a lot of studies about the effects of N deposition on Rs, the results remain controversial. For example, Wang *et al*.^14^ reported that N addition increased the Rs due to the enhanced C availability on Chinese Loess Plateau. However, Janssens *et al*.^15^ reported that N might be limiting microbial growth and thus decreased Rs. Furthermore, Eberwein *et al*.^16^ observed that N addition increased Rs when C was abundant, while decrease Rs when C was limited. The inconsistency of these results might reflect study differences in ecosystem type, nutrient limitation and the rate of N addition^17,18^. In addition, the difference in results is partly owing to Rs which is a multisource flux, usually partitioned into the autotrophic respiration (Ra) and heterotrophic respiration (Rh)^19^. Generally, Ra is the sum of root and rhizosphere microbial respiration, which is driven by belowground C allocation^20^. Rh is derived from the decomposition of litter and organic matter, which is affected by the microbial activity and substrate concentration^20^. N fertilizer may improve Ra by promoting plant growth. Meanwhile, N fertilizer may suppress Ra through reduce photosynthesis allocated belowground^19^. Similarly, N fertilizer may improve Rh by enhancing soil microbial biomass and microbial activity or suppress it through reduce enzyme activity and decomposition of soil organic matter^19^. In addition, soil temperature, soil moisture content^21,22^, and C and N pools^23,24^ are also important factors that regulate Rs by regulating respiratory enzyme activity and substrate supplies. However, it is still unclear how these variables directly or indirectly control autotrophic and heterotrophic respiration. Therefore, further studies should be conducted to understand the main factors driving Ra and Rh for further C balance management.

Previous studies about N fertilizer on soil respiration have been conducted in various ecosystems^16,18,19,25,26^. However, the results of N-addition on respiration components were not consistent. For example, a study conducted in a Tibetan alpine steppe showed that N enrichment increased Rs and Ra but decreased Rh^26^. Zhang *et al*.^25^ showed that the growing season Rs, Ra and Rh were significantly increased in high N-addition treatment (92 kg N ha^−2^ y^−1^) in semiarid grassland. Chen *et al*.^19^ reported that N supply significantly increased the Rs and Ra, but had no effects on Rh in a rain fed cropland ecosystem. However, a study conducted by Olsson *et al*.^27^ showed that N fertilizer decreased Rs and Ra in a forest ecosystem. Therefore, further study about the responses of Ra and Rh to N supply is vital in accurately predicting future global C balance.

N deposition and rising atmospheric carbon dioxide content are two main mechanisms underlying terrestrial ecosystems C sequestration^28^. Therefore, quantifying the magnitude of Rs and C storage in response to different levels of N application in grassland ecosystem can help build a model projection of future climate change. However, how the increasing N deposition influences C cycles remains unclear^29,30^. In addition, it remains unclear how N addition influences Rs and, more specifically, how N deposition influences Rs by affecting C storage. Given the above issues, in this research, we conducted a three-year field experiment that simulated atmospheric N deposition in alpine meadow region on the northeast margin of Qinghai-Tibetan Plateau to elucidate the impacts of N deposition on Ra, Rh and Rs, C and N storage. The purpose of our study was: (1) to identify the response of Rs and its components to various gradients of N addition, (2) to investigate the responses of vegetation and soil C and N storage to various gradients of N addition, (3) to explore the mechanisms by which N deposition influences Rs by affecting C and N storage. The ultimate goal of this research was to explore the underlying responses of ecosystem C storage and CO_2_ fluxes to N addition.

## Results

### Biotic and abiotic variables

During the two experimental years, the above- and below-ground biomass were higher in the N-addition treatments than N0 treatment. Meanwhile, the above- and below-ground biomass showed an increasing trend with increasing amounts of N addition up to the N5 treatment, and then decreased in the N6 treatment (Table 1). N application had no significant impact on litter biomass and soil bulk density in 0–10, 10–20 and 20–30 cm soil layer during the two experimental years. In 2014 and 2015, the soil temperature at 5 cm depth (ST5), soil moisture content at 10 cm depth and (SM10) soil microbial biomass carbon (SMBC), soil organic carbon (SOC) and soil total nitrogen (TN) contents were significantly increased in the N3, N4, N5 and N6 treatments relative to N0 treatment.

**Table 1:** The biotic and abiotic variables after 2 and 3 years of nitrogen-addition (2014 and 2015, respectively) in an alpine meadow ecosystem on the northeast margin of the Qinghai-Tibetan Plateau.

Variable	Year	Nitrogen addition treatments						
		N0	N1	N2	N3	N4	N5	N6
ABG (g•m^−2^)	2014	380.47 ± 12.65e	468.54 ± 17.81d	529.33 ± 14.55c	576.40 ± 13.28b	623.65 ± 16.74a	647.28 ± 17.89a	544.40 ± 10.42bc
	2015	349.95 ± 17.85 f	458.22 ± 26.22e	521.23 ± 12.56d	596.87 ± 15.55c	687.57 ± 18.24b	782.29 ± 17.14a	709.37 ± 18.25b
BGB (g•m^−2^)	2014	3411.06 ± 138.56c	4377.60 ± 115.43b	4519.88 ± 99.78b	5289.64 ± 115.47a	5617.37 ± 196.33a	5568.58 ± 190.75a	4577.19 ± 173.21b
	2015	3257.14 ± 138.25d	3586.74 ± 167.65cd	3924.6 ± 201.55c	4659.88 ± 201.57b	5351.58 ± 187.52a	5624.97 ± 284.55a	4837.19 ± 188.56b
LB (g•m^−2^)	2014	33.17 ± 2.89a	32.46 ± 3.46a	33.22 ± 2.91a	33.28 ± 2.31a	34.02 ± 2.31a	33.23 ± 3.46a	32.33 ± 0.88a
	2015	34.48 ± 2.31a	33.97 ± 1.73a	33.78 ± 1.73a	35.16 ± 2.89a	34.96 ± 2.31a	35.33 ± 0.88a	35.07 ± 2.89a
Shannon-Wiener diversity index	2014	2.52 ± 0.03a	2.53 ± 0.04a	2.46 ± 0.02a	2.21 ± 0.03abc	1.88 ± 0.02c	2.18 ± 0.02b	2.24 ± 0.03ab
	2015	2.48 ± 0.03a	2.45 ± 0.03a	2.41 ± 0.02a	2.26 ± 0.03ab	2.05 ± 0.03bc	1.76 ± 0.02c	1.84 ± 0.02c
Pielou evenness index	2014	0.97 ± 0.01a	0.96 ± 0.01a	0.89 ± 0.01ab	0.87 ± 0.01ab	0.84 ± 0.01ab	0.80 ± 0.01b	0.84 ± 0.01ab
	2015	0.95 ± 0.02a	0.91 ± 0.01ab	0.86 ± 0.01ab	0.82 ± 0.01ab	0.77 ± 0.01ab	0.71 ± 0.01b	0.76 ± 0.01ab
Richness index	2014	14.00 ± 0.00a	14.00 ± 0.00a	13.67 ± 0.33a	13.00 ± 0.00a	12.67 ± 0.54a	12.67 ± 0.69a	12.67 ± 0.53a
	2015	13.67 ± 0.76a	13.33 ± 0.46a	13.33 ± 0.52a	13.00 ± 0.00a	12.67 ± 0.49a	12.33 ± 0.38a	12.67 ± 0.49a
ST5 (°C)	2014	10.73 ± 0.22a	10.69 ± 0.24a	10.71 ± 0.24a	10.29 ± 0.21b	10.17 ± 0.2b	9.97 ± 0.19b	10.11 ± 0.2b
	2015	10.86 ± 0.19a	10.57 ± 0.17ab	10.31 ± 0.16b	10.41 ± 0.15b	10.23 ± 0.12b	10.14 ± 0.13b	10.13 ± 0.13b
SM10 (%)	2014	26.62 ± 0.69b	26.82 ± 0.71b	26.77 ± 0.69b	27.15 ± 0.68a	27.13 ± 0.68a	27.19 ± 0.68a	27.09 ± 0.7a
	2015	25.53 ± 0.95b	25.61 ± 0.93b	25.34 ± 0.95b	25.67 ± 0.95b	26.16 ± 0.9a	26.29 ± 0.98a	26.16 ± 0.91a
SOC in 0–10 cm layer (g•kg^−1^)	2014	46.23 ± 1.27d	46.67 ± 0.3d	49.11 ± 0.65c	50.37 ± 0.35bc	51.82 ± 0.51b	53.94 ± 0.33a	49.69 ± 0.51c
	2015	46.54 ± 1.87d	46.98 ± 0.62cd	47.18 ± 0.19cd	49.48 ± 0.2bc	51.24 ± 0.51b	55.49 ± 0.51a	50.27 ± 0.19b
SOC in 10–20 cm layer (g•kg^−1^)	2014	35.11 ± 0.65b	34.92 ± 0.54b	35.09 ± 0.69b	36.60 ± 0.55b	35.56 ± 0.23b	39.33 ± 0.56a	38.98 ± 0.64a
	2015	36.12 ± 0.69c	36.19 ± 0.97c	34.65 ± 0.11c	36.16 ± 0.34c	37.92 ± 0.26b	40.8 ± 0.51a	38.82 ± 0.49b
SOC in 20–30 cm layer (g•kg^−1^)	2014	25.68 ± 0.05a	25.46 ± 0.29a	25.56 ± 0.32a	24.86 ± 0.29a	26.24 ± 1.03a	26.35 ± 0.26a	25.85 ± 0.47a
	2015	26.39 ± 0.88a	26.80 ± 0.39a	25.02 ± 0.6a	25.68 ± 0.88a	24.94 ± 0.44a	26.10 ± 0.33a	26.26 ± 0.31a
TN in 0–10 cm layer (g•kg^−1^)	2014	4.20 ± 0.01e	4.19 ± 0.01e	4.33 ± 0.02d	4.44 ± 0.01c	4.57 ± 0.01b	4.72 ± 0.03a	4.53 ± 0.03b
	2015	4.24 ± 0.03e	4.22 ± 0.01e	4.36 ± 0.02d	4.44 ± 0.01c	4.60 ± 0.06b	4.80 ± 0.01a	4.60 ± 0.01b
TN in 10–20 cm layer (g•kg^−1^)	2014	3.67 ± 0.02e	3.72 ± 0.01de	3.71 ± 0.02de	3.79 ± 0.02cd	3.90 ± 0.03ab	3.95 ± 0.05a	3.85 ± 0.02bc
	2015	3.71 ± 0.04e	3.69 ± 0.01e	3.71 ± 0.02e	3.81 ± 0.01cd	3.89 ± 0.02b	3.94 ± 0.03a	3.84 ± 0.01b
TN in 20–30 cm layer (g•kg^−1^)	2014	3.46 ± 0.03a	3.49 ± 0.01a	3.48 ± 0.01a	3.49 ± 0.01a	3.41 ± 0.01a	3.44 ± 0.02a	3.45 ± 0.01a
	2015	3.45 ± 0.01a	3.43 ± 0.01a	3.47 ± 0.01a	3.46 ± 0.01a	3.43 ± 0.01a	3.45 ± 0.02a	3.47 ± 0.01a
MBC in 0–10 cm layer (mg•kg^−1^)	2014	399.43 ± 10.77c	406.05 ± 9.5c	403.84 ± 8.61c	436.25 ± 11.91b	433.57 ± 7.36b	468.99 ± 9.52a	423.46 ± 9.36bc
	2015	379.53 ± 9.03d	393.07 ± 7.28cd	393.43 ± 7.0 cd	414.88 ± 9.6bc	426.55 ± 11.53b	456.81 ± 9.2a	431.93 ± 12.16b
BD in 0–10 cm layer (g•cm^−3^)	2014	0.73 ± 0.03a	0.76 ± 0.04a	0.74 ± 0.04a	0.75 ± 0.07a	0.76 ± 0.05a	0.75 ± 0.07a	0.76 ± 0.1a
	2015	0.72 ± 0.02a	0.73 ± 0.03a	0.72 ± 0.03a	0.72 ± 0.06a	0.73 ± 0.03a	0.71 ± 0.02a	0.71 ± 0.01a
BD in 10–20 cm layer (g•cm^−3^)	2014	0.80 ± 0.03a	0.81 ± 0.03a	0.81 ± 0.02a	0.80 ± 0.05a	0.81 ± 0.02a	0.79 ± 0.01a	0.80 ± 0.01a
	2015	0.80 ± 0.02a	0.81 ± 0.03a	0.80 ± 0.03a	0.81 ± 0.03a	0.81 ± 0.04a	0.81 ± 0.04a	0.80 ± 0.02a
BD in 20–30 cm layer (g•cm^−3^)	2014	0.82 ± 0.03a	0.82 ± 0.03a	0.82 ± 0.02a	0.82 ± 0.04a	0.82 ± 0.04a	0.83 ± 0.01a	0.83 ± 0.02a
	2015	0.84 ± 0.03a	0.85 ± 0.03a	0.84 ± 0.03a	0.83 ± 0.05a	0.84 ± 0.06a	0.84 ± 0.02a	0.86 ± 0.02a

Note: N0, N1, N2, N3, N4, N5 and N6 represent 0, 54, 90, 126, 144, 180 and 216 kg N ha^−1^ yr^−1^, respectively. Data are showed as the means ± standard error. Different letters in the same row indicate significant differences at *P* < 0.05. The belowground biomass was calculated for the 0–30 cm depth. ABG: Aboveground biomass; BGB: Belowground biomass; LB:Litter biomass; MBC: Microbial biomass carbon; SM10: Volumetric soil moisture content at 10 cm depth; ST5: Soil temperature at 5 cm depth; SOC: Soil organic carbon; TN: Soil total nitrogen; BD: Soil bulk density.

### Soil respiration and its components

**Figure 1:**
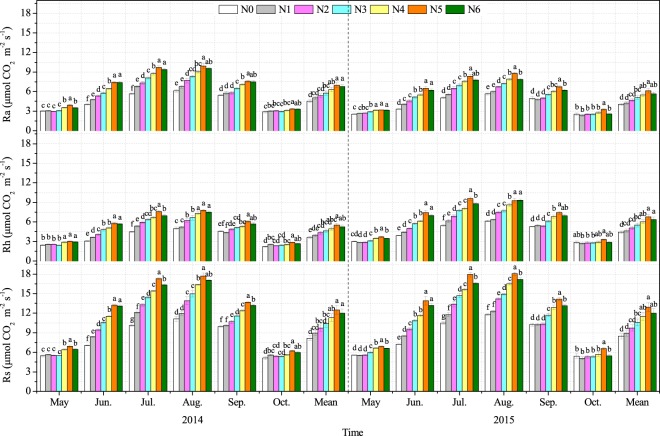
Seasonal variation in autotrophic respiration (Ra), heterotrophic respiration (Rh) and soil respiration (Rs) in an alpine meadow under different nitrogen-addition treatments. N0, N1, N2, N3, N4, N5 and N6 represent 0, 54, 90, 126, 144, 180 and 216 kg N ha^−1^ yr^−1^, respectively. Mean: the average value of soil respiration from May to October. Different letters in the same month indicate significant differences at *P* < 0.05. Twelve replications for Rs, Ra and Rh at each month, respectively.

During the two experimental years, the Ra, Rh and Rs showed similar seasonal variation among the various N-addition treatments, with the higher values found in August and lower values found in October (Fig. 1). Meanwhile, during the growing seasons (May - October), the Ra, Rh and Rs showed an increasing trend with increasing amounts of N-addition up to the N5 treatment, and then decreased in the N6 treatment. The growing seasons mean Ra, Rh and Rs were significantly increased in the N3, N4, N5 and N6 treatments in both experimental years compared to the N0 treatment (Fig. 1).

### Carbon and nitrogen storage

**Figure 2:**
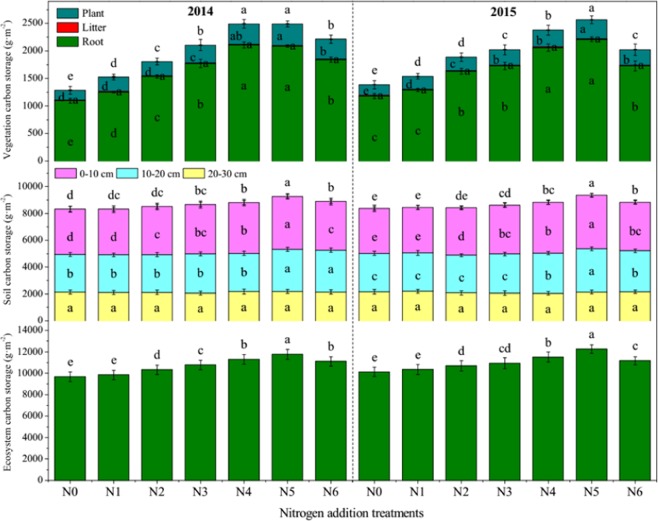
The organic carbon storage in vegetation, soil and ecosystem in an alpine meadow under different nitrogen addition treatments. N0, N1, N2, N3, N4, N5 and N6 represent 0, 54, 90, 126, 144, 180 and 216 kg N ha^−1^ yr^−1^, respectively. Different letters indicate significant differences at *P* < 0.05.

**Figure 3:**
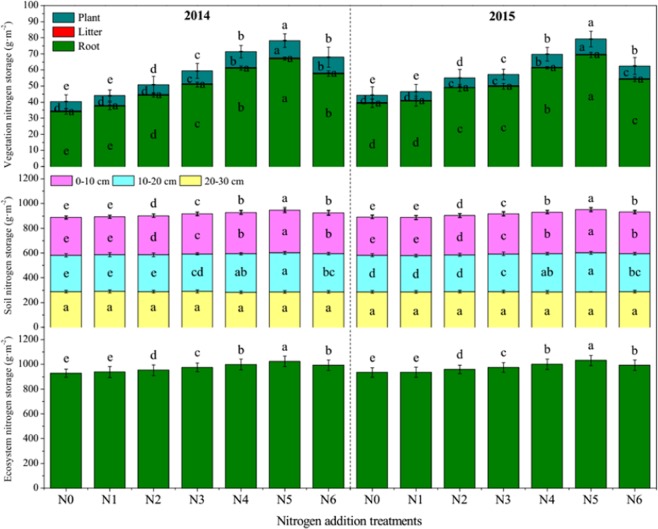
The total nitrogen storage in vegetation, soil and ecosystem in an alpine meadow under different nitrogen addition treatments. N0, N1, N2, N3, N4, N5 and N6 represent 0, 54, 90, 126, 144, 180 and 216 kg N ha^−1^ yr^−1^, respectively. Different letters indicate significant differences at *P* < 0.05.

The C storage showed an increasing trend in plant and root with increasing amounts of N addition up to the N5 treatment, and then decreased in the N6 treatment. However, N addition had no impact on litter C or N storage. The vegetation C storage was significantly increased in the N application treatments compared to the N0 treatment in 2014 and 2015. N storage in plant, litter and root showed similar trends to those of C storage. At 0–10 and 10–20 cm soil layer, SOC storage and total N storage differed significantly in the different N-addition treatments, whereas in deeper soil, N application had no significant impact on SOC or N storage (Figs 2 and 3). The SOC storage in the 0–30 cm soil layer was significantly increased in the N3, N4, N5 and N6 treatments in both experimental years (Fig. 2). While the soil total N storage in the 0–30 cm soil layer was significantly increased in the N2, N3, N4, N5 and N6 treatments in both experimental years. The ecosystem C and N storage all showed an increasing trend with increasing amounts of N-addition up to the N5 treatment, and then decreased in the N6 treatment (Figs 2 and 3). Ecosystem C storage and N storage were significantly increased in all of the N-addition treatments except the N1 treatment in both experimental years.

### Soil respiration and its components in relation to carbon and nitrogen storage

**Figure 4:**
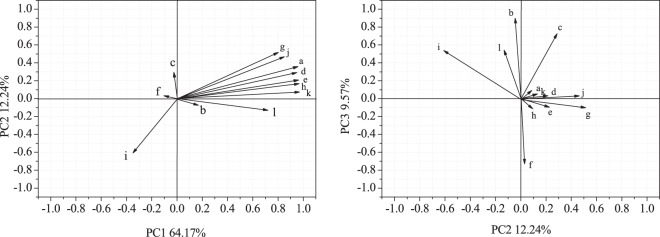
Principal component analysis of soil respiration as explained by carbon and nitrogen storage. a-soil organic carbon storage in the 0–10 cm soil layer, b-soil organic carbon storage in the 10–20 cm soil layer, c-soil organic carbon storage in the 20–30 cm soil layer, d-soil nitrogen storage in the 0–10 cm soil layer, e-soil nitrogen storage in the 10–20 cm soil layer, f-soil nitrogen storage in the 20–30 cm soil layer, g-plants carbon storage, h-root carbon storage in 0–30 cm soil layer, i-litter carbon storage, j-plants nitrogen storage, k-root nitrogen storage in 0–30 cm soil layer, l-litter nitrogen storage.

A principal component analysis (PCA) was used to determine the correlations among the C and N storage in plant, litter and soil (Fig. 4). The results showed that the first, second, and third axes explained 64.17%, 12.24% and 9.57% of the standardized variance, respectively. The highly weighted parameters (loading value > 0.900) on PC1 were soil C storage in the 0–10 cm layer, soil N storage in the 0–10 and 10–20 cm layers, and root C and N storage. The highly loaded parameter for PC3 was soil C storage in the 10–20 cm layer.

**Figure 5:**
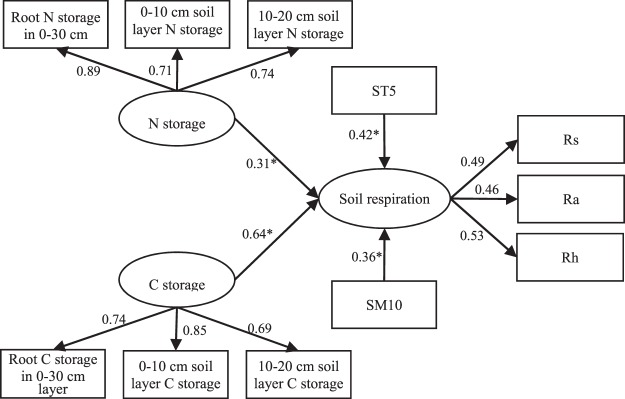
Structural equation model describing the influences of carbon and nitrogen storage, soil temperature at 5 cm depth and soil moisture content at 10 cm depth on growing season soil respiration and their relationships among each other. The rectangular boxes denote the observed variables, the ellipses denote latent variables. One-headed arrows indicate the relationships between variables. Numbers at arrows indicate standardized path coefficients or covariation coefficients. ST5: soil temperature at 5 cm depth; SM10: soil moisture content at 10 cm depth; Rs: total soil respiration; Ra: autotrophic respiration; Rh: heterotrophic respiration. *Indicates a significant difference at the 0.05 level.

**Figure 6:**
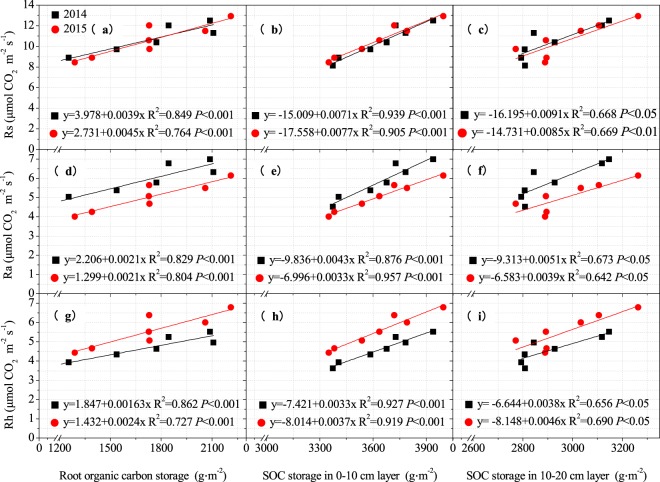
The relationships between soil respiration components (Rs, soil respiration; Ra, autotrophic respiration; Rh, heterotrophic respiration) and organic carbon storage.

**Figure 7:**
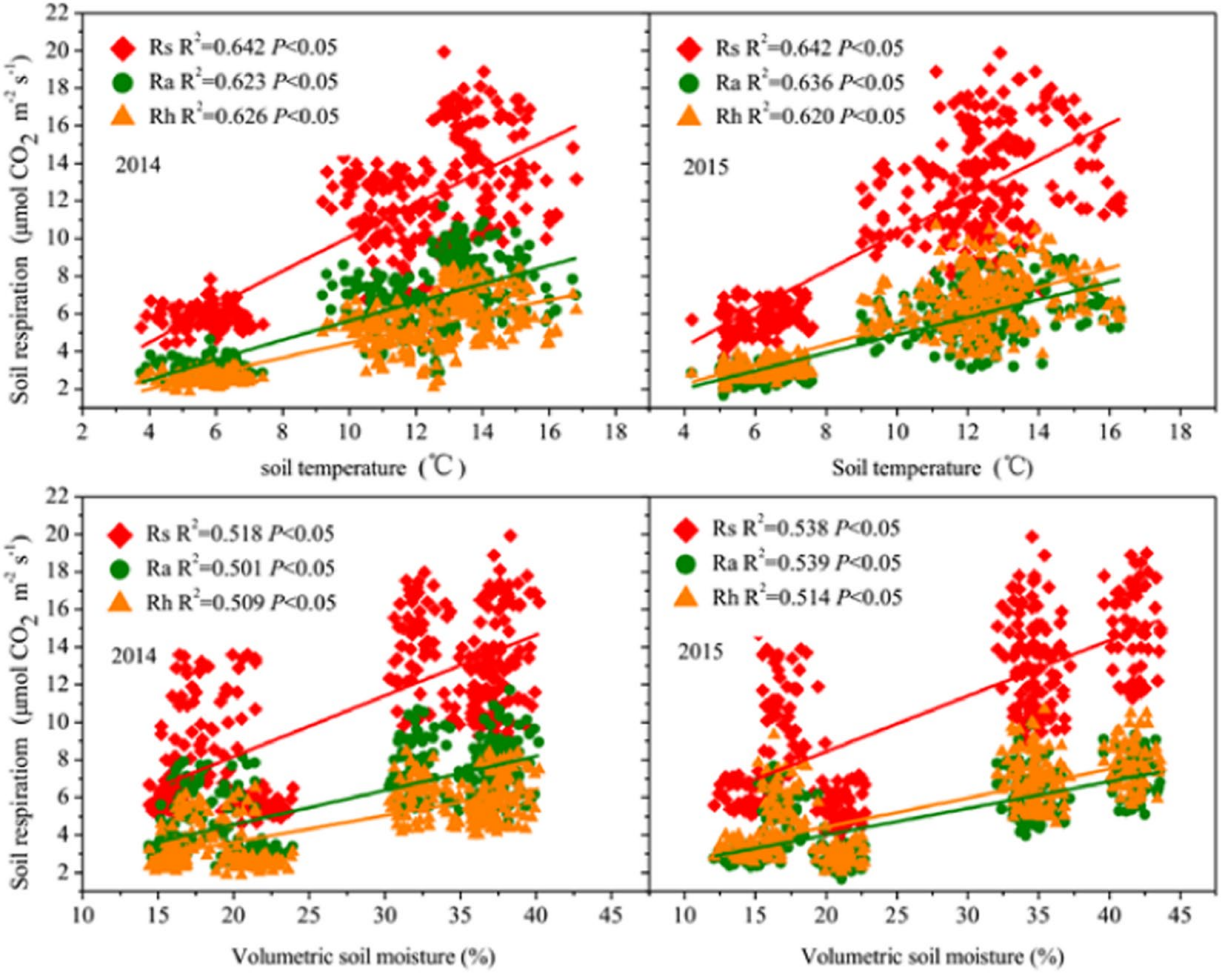
The relationships between soil respiration (Rs); autotrophic respiration (Ra); heterotrophic respiration (Rh) and soil temperature and volumetric soil moisture.

We chose the parameters with loading value greater than 0.900 in the PCA results, ST5 and SM10 as predictors to establish structural equation modelling (SEM) to explore the impact of C and N storage, soil temperature and soil moisture content on growing season Rs (Fig. 5). The results explained the changes in Rs well (goodness of fit index was 0.752; χ^2^ = 7.99 and *P* = 0.18). The results showed that C storage had significant effect on growing season Rs (*P* < 0.05). While N storage, soil temperature and soil moisture had no significant effects on growing season Rs (*P* > 0.05). Simultaneously, the path coefficient of ST5 on Rs (0.42) was higher than the path coefficient of SM10 on Rs (0.36). Therefore, we concluded that C storage (in root, the 0–10 and 10–20 cm soil depths) is the most predominant variable controlling growing season soil respiration. Meanwhile, ST5 has a stronger effect than SM10 on soil respiration. Rs, Ra and Rh had positive linear relationships with root C storage, 0–10 cm soil layer C storage, 10–20 cm soil layer C storage (Fig. 6), soil temperature and soil moisture (Fig. 7). Multiple linear regression model showed that root carbon storage, soil organic carbon storage in 0–10 cm layer and soil organic carbon storage in 10–20 cm layer explained more than 94.5% of Rs, Ra and Rh (Table 2).

**Table 2:** Multiple linear regression equations of soil respiration (Rs), autotrophic respiration (Ra) and heterotrophic respiration (Rh) against root carbon storage (RCS), soil organic carbon storage in 0–10 cm layer (SOCS_10_), soil organic carbon storage in 10–20 cm layer (SOCS_20_), volumetric soil moisture at 10 cm depth (SM10) and soil temperature at 5 cm depth (ST5).

	Year	Equation	R^2^	*P*-value
Rs	2014	Rs = 0.003*RCS-0.002*SOCS_10_ + 0.006*SOCS_20_-3.344	0.974	0.005
		Rs = 0.139*SM10 + 0.659*ST5-0.231	0.690	0.008
	2015	Rs = 0.006*RCS-0.009*SOCS_10_ + 0.011*SOCS_20_ + 2.356	0.976	0.002
		Rs = 0.144*SM10 + 0.707*ST5-0.686	0.713	0.007
Ra	2014	Ra = 0.003*RCS-0.004*SOCS_10_ + 0.004*SOCS_20_ + 4.072	0.960	0.005
		Ra = 0.078*SM10 + 0.373*ST5–0.18	0.669	0.004
	2015	Ra = 0.002*RCS-0.003*SOCS_10_ + 0.005*SOCS_20_-0.680	0.945	0.008
		Ra = 0.07*SM10 + 0.335*ST5-0.351	0.710	0.003
Rh	2014	Rh = 0.0001*RCS + 0.002*SOCS_10_ + 0.002*SOCS_20_-7.417	0.963	0.004
		Rh = 0.062*SM10 + 0.286*ST5-0.051	0.675	0.000
	2015	Rh = 0.004*RCS-0.006*SOCS_10_ + 0.006*SOCS_20_ + 3.036	0.981	0.002
		Rh = 0.074*SM10 + 0.372*ST5-0.334	0.686	0.000

## Discussion

### Soil respiration and its components

During the two experimental years, the growing season Rs was significantly increased in the N-addition treatments, which suggests the stimulate effects of N fertilizer on Rs in alpine meadow ecosystem. Similar results were found in alpine steppe^26^ and semiarid grassland ecosystem^25,30^. Meanwhile, N-addition significnatly stimulated the growing season Ra and Rh during the two experimental years. Therefore, we concluded that the stimulated effect of N-addition on Rs was mainly due to the increase of Rh and Ra in alpine meadow on the northeastern Qinghai-Tibetan Plateau. The alpine meadow is an N limited grassland ecosystem and has strong capacity to immobilize external N^31^. Therefore, the above- and below-ground biomass was significantly increased after N-addition. This can increase the supply of C substrates to support root and microbial internal stoichiometric requirements and thereby increase Rh^32^. Generally, root activity and biomass are major factors driving Ra^33,34^. Soil C concentration is an important regulator of Rh^21^. The N-addition increases the soil N availability and thus promotes the plants growth in grassland^31^. The increased soil N content may meet the demand of soil microorganisms for N source, thus promoting microbial biomass and activity. Our study showed that the above- and below-ground biomass was significantly increased in the N2-N6 treatments relative to the N0 treatment (Table 1). Larger root biomass represents more root surface area and associated with higher Ra^35^. Therefore, the increased root biomass may improve the Ra. Previous studies also indicated that N-addition increased the root biomass and Ra^25,36^. However, a privious study conducted in a artificial forest showed that 8 years N-addition had no impact on Rs^37^. They interpreted this due to a decrease in microbial biomass after N-addition. Generally, N fertilizer could increase the N source for soil microorganism, and thus increased the microbial biomass and activity. In the current study, the microbial biomass carbon was significantly increased in N3-N5 treatments during the two experimental years (Table 1). The increased microbial biomass may improve the Rh and thereby increase Rs. Compared to the N5 treatment, the mean Rs in the N6 treatment was decreased by 4.0% and 6.9% in 2014 and 2015, respectively. In the current study, root biomass showed a similar hump-shape pattern response to Rs with increasing level of nitrogen addition (Table 1). Before the N saturation point (180 kg N ha^−1^ yr^−1^), root biomass increased, whereas after the saturation point, root biomass decreased. Meanwhile, the high-level N addition (>180 kg N ha^−1^ yr^−1^) can lead to soil acidification^38^, this in turn will accumulate poisonous substances and inhibit the activities of soil microorganisms^39^. Our research showed that the topsoil microbial biomass carbon in the N6 treatment was significnatly decreased by 9.7% and 5.4% in 2014 and 2015, compared to the N5 treatment. Thus, the decreased root biomass and microbial biomass carbon in the N6 site may decrease the root respiration and microbial respiration, and ultimately decrease the soil respiration.

### Carbon and nitrogen storage

N-addition increased the content of soil available nitrogen, and thus stimulated the plant growth, and the plant C and N storage increased as a result. The experimental site was grazed in the cold season (November to May) by Tibetan sheep at a high livestock density (6.67–7.22 head per hectare). Almost all the above-ground biomass was removed by the herbivores until May of the next year. There was only little litter (33–38 g/m^2^) in the experimental site. Meanwhile, there was no significant difference in litter biomass or litter C and N contents among the N-addition treatments. Therefore, N application had no significant impact on litter C or N storage in current study. Previous researches about the effect of N enrichment on soil C storage were still highly controversial. For example, N addition significantly increased the 0–10 cm layer SOC storage in a Europe forest ecosystem^40^. However, some studies reported that N application significantly decreased the SOC storage^41,42^. In these studies, N additions decreased the root biomass which led to little C allocation to belowground, and thus the SOC pool was decreased. In our study, the C storage in the 0–30 cm soil layer increased by 0.6–11.8% in N-addition treatments. Wang *et al*.^43^ reported that the 0–40 cm layer SOC content increased by 34.7% after two years of N addition (200 kg N ha^−1^ yr^−1^) in an alpine meadow on the Qinghai-Tibetan Plateau. Dong *et al*.^44^ found that the SOC storage in 0–60 cm soil layer increased by 21.7% after 15 years of N addition (200 kg N ha^−1^ yr^−1^) in a farmland ecosystem of the North China Plain. Generally, SOC storage comes from photosynthetic carbon fixation. Plant root is crucial in controlling SOC pool through root decomposition and exudation. In our study, N addition caused a surge of dominant *Elymus nutans*, which have relatively greater abundance of leaf area. During the growing seasons, the plentiful sunlight makes the photosynthesis stronger in the N application treatments^45^. Therefore, N addition should stimulate root activity and increase root biomass^46^, thereby increasing soil C and N storage. In addition, the soil temperature difference between day and night is large in alpine meadow on the Qinghai-Tibetan Plateau^47^. The low temperature in the night may restrict the microbial and root activity that limited Ra and Rh^48^. Therefore, the high photosynthetic carbon fixation and low decomposition of organic matter made the higher SOC storage in the N application treatments in alpine meadow ecosystem on the Qinghai-Tibetan Plateau.

N addition influences the composition and structure of the vegetation community (Table 1) and thus influences ecosystem C and N storage^49^. Generally, C and N storage increased with increasing N-addition rate in the current study. However, relative to the N5 level, the highest level of N addition (216 kg N ha^−1^ yr^−1^) decreased the vegetation, soil and ecosystem C and N storage. These results indicate that C and N storage appears to saturate at a high rate of N addition (216 kg N ha^−1^ yr^−1^) in excess of the N saturation point (180 kg N ha^−1^ yr^−1^). In cold region, temperature is the most important factor limiting plant growth^36^. Plants may have a threshold of nitrogen uptake in alpine meadow due to the low temperature, above which plant may not absorb more nitrogen even in high N-addition treatment. Furthermore, the high rate of N addition can lead to soil acidification, which in turn will limit plant and root growth, and thus decrease C and N storage in plant and root^36,50,51^. Our research also indecated that the above- and below-ground biomass in the N6 treatment were signifcantly decreased by 15.9% and 9.3%, respectively, compared to the N5 treatment. The decomposition of dead root and litter is the main source of soil organic matter^24^. The decreased plant and root biomass in the N6 site may decrease the vegetation and soil C and N storage. Accordingly, high N addition beyond the N saturation point (180 kg N ha^−1^ yr^−1^) decreased vegetation, soil and ecosystem C and N storage.

### Correlations between soil respiration and its components and each of C and N storage

Previous researches about the factors driving Rs have mainly focused on ST, SM, plant, litter and below-ground biomass^52,53^; and soil C and N concentration^18^. Generally, ST and SM are two key abiotic factors that control daily and seasonal variation in Rs across different vegetation types^54^. Previous study in a Tibetan Kobresia pastures found that ST5 had a stronger impact than did SM10 on growing season Rs^22^. However, studies conducted in temperate grassland and steppe grassland reported that SM10 had a stronger impact than did ST5 on growing season Rs^24,55,56^. In the present study, the SEM analysis revealed that soil temperature had a stronger effect than did soil moisture on Rs during the growing seasons. Soil temperature and moisture affect respiration by affecting respiratory enzymatic activity and substrate supply^57^. Generally, low temperature and soil water content limit the enzymatic activity and diffusion of soluble substrates; while high temperature and soil moisture also limit the enzymatic activity and the diffusion of oxygen, thus, both of which can suppress Rs^57^. Therefore, appropriate soil temperature and soil moisture are essential for soil respiration. Yan *et al*.^58^ reported that there was a threshold (14%) at which the effects of volumetric soil water content on Rs changed, above which soil moisture is not the limiting factor of Rs^58^. Meanwhile, a previous study indicated that there was a threshold at which the effects of volumetric soil water content on soil respiration changed; that is, the two were positively correlated at soil water contents of 30% or lower, and negatively correlated at soil water contents of >30%^59^. In our study, the volumetric soil moisture in growing season was 25.3–27.2%, which is no longer the factor that influences the diffusion of soluble substrates. Furthermore, the alpine meadow on the Qinghai-Tibetan Plateau is a temperature limited ecosystem^60^. The mean ST5 of the growing season was between 10.1°C and 10.9°C, which may be below the threshold of soil temperature, and thus limit the respiratory enzymatic activity.

Although soil temperature and soil moisture had important effect on Rs, some previous researches indicated that some biotic parameters might have stronger effects on the Rs rate than do either SM10 or ST5^23,61^. For example, previous studies showed that root and soil carbon storage significantly control the Rs through manipulating Ra and Rh in forest ecosystem^23,62^. Li *et al*.^24^ also revealed that the root and topsoil C pools were two most important factor that controlling the Rs in an alpine meadow on the Qinghai-Tibetan Plateau. In our study, the SEM results showed that C storage (in root, 0–10 and 10–20 cm soil depths) have greater influences on Rs than do ST5 or SM10 (Fig. 5). Indicating that root C storage and C storage in topsoil (in 0–10 and 10–20 cm soil depth) are two important factors driving Rs. This mainly attribute to the impacts of belowground C storage on the production of detritus and exudates for soil microbial activities^23^. C storage is a complex parameter representing the vegetation biomass and C concentration. The increase in belowground C storage was related to an increase in root biomass and thus increased Rs. We found that N-addition significantly increased the root and soil carbon storage (Fig. 2). General linear regression analysis indicated that growing season Rs, Ra and Rh had significant positive correlation with root carbon storage and soil carbon storage (Fig. 6). The reason for this was the increased soil and root C storage that can meet the microbial demand for C source, and thereby increased Rh. Meanwhile, the combined root carbon storage, 0–10 cm and 10–20 cm soil layer carbon storage explained 97.4–97.6% variations in Rs; explained 94.5–96% variations in Ra; and explained 96.3–98.1% in Rh, which was higher than the combined soil temperature and soil moisture did (Table 2). Therefore, the growing season soil respiration and its components can be well predicted by the root and topsoil organic C storage in alpine meadow of the north-eastern Qinghai-Tibetan Plateau.

## Conclusion

Short-term N-addition can significantly increase the soil respiration and its components, C and N storage in plant, root and topsoil (0–10 cm and 10–20 cm soil layer). Meanwhile, our results revealed that the stimulated effect of N-addition on Rs was mainly due to the increase of Rh and Ra. In addition, the ecosystem C and N storage appears to saturate at a high rate of N addition (216 kg N ha^−1^ yr^−1^) in excess of the N saturation point (180 kg N ha^−1^ yr^−1^). However, the current study was a short term experiment, and how Rs and C storage respond to the long-term N-addition remains unclear. Therefore, further studies should consider these for better understanding of the impacts of increasing N enrichment on C cycling in grassland ecosystem. The SEM results showed that C storage in root, 0–10 and 10–20 cm soil depths have greater influences on soil respiration than do soil temperature or soil moisture. Meanwhile, multiple linear regression model showed the combined root carbon storage, topsoil carbon storage explained 97.4–97.6% variations in Rs; explained 94.5–96% variations in Ra; and explained 96.3–98.1% in Rh, which was higher than the combined soil temperature and soil moisture did. Therefore, the growing season soil respiration and its components can be well predicted by the root and topsoil organic C storage in alpine meadow of the north-eastern Qinghai-Tibetan Plateau. The SEM results also indicated that soil temperature had a greater impact on growing season soil respiration than did volumetric soil moisture. Considering productive functions and ecosystem C storage, the optimum level of N fertilizer is 180 kg N ha^−1^ yr^−1^ in alpine meadow of the northeast margin of Qinghai-Tibetan Plateau.

## Materials and Methods

### Study area

The research was performed in an alpine meadow region of the northeast margin of Qinghai-Tibetan Plateau located in Zhuaxixiulong Township, Tianzhu Tibetan Autonomous County, Gansu Province, PR China (37°11′N, 102°46′E; 2960 m a.s.l.). The area is approximately 180 km northwest of Lanzhou, PR China. The mean annual temperature in the study area was 0.13 °C (varying from −11.4 °C in January to 11.2 °C in July) and mean annual precipitation was 414.98 mm, approximately 63.3–88.2% of the rainfall falling between June and September (climatological data from 1951 to 2016)^63^. In this region, the vegetation growing season is from May to October. The soil in the study area belongs to alpine chernozem. The soil organic carbon and total nitrogen content of the 0–10 cm soil layer at the beginning of the study ranged from 47.6 to 49.9 g•kg^−1^ and 4.2 to 4.4 g•kg^−1^, respectively. The alpine meadow is dominated by *Elymus nutans*, *Poa crymophila* And *Kobresia humilis*.

### Experimental design

In the middle of June 2013, we established three blocks (18 × 138 m per block) as replicates. All blocks were located 10 m away from each other. Each block contained seven plots (18 m × 18 m per plot), with 2 m of walkway established between contiguous plots. N fertilizer was added as urea (CO(NH_2_)_2_, 46.6% N). The highest rate of N deposition that had occurred previously in the study area was 22.6 kg N ha^−1^ in 2010, and the annual rate of increase in N deposition in this region was 0.42 kg N ha^−1^ yr^−1^ ^64,65^. Therefore, we chose seven N-addition treatments in this study: 0 (N0), 54 (N1), 90 (N2), 126 (N3), 144 (N4), 180 (N5) and 216 kg N ha^−1^ yr^−1^ (N6). The study was done using a randomized complete block design, and all of the N applications were randomly assigned to the seven plots of each block. The N fertilizer was evenly spread by hand in each plot in early July 2013, 2014 and 2015 following rainfall events. Our experiment was conducted from May to October in 2013, 2014 and 2015, respectively. The experimental grassland served as a local winter pasture; it was grazed from November to May by Tibetan sheep (6.67–7.22 head per hectare) and was fenced the remainder of the year.

### Field measurements and sampling

#### Soil respiration and its components

In late May of 2013, four intact, untrenched subplots (1 m × 1 m) were randomly established in each plot. Meanwhile, we excavated a trench (60 cm diameter × 70 cm deep, no root deeper than 70 cm) on the outside edges of each untrenched subplot. For a total of twelve untrenched and trenched subplots of each N addition treatment, respectively. The location of each untrenched and trenched subplots were at least 1 m away from the margin to avoid edge effects. The trenches were lined with a nylon mesh bag (0.038 mm mesh, 60 cm diameter × 75 cm deep) to prevent root growth into the plots (but allow the movement of water, microorganism, organic matter and minerals). We removed the root using a 2-mm sieve and then refilled the trenches with the root-free soil layer by layer^25,66^. Therefore, the CO_2_ efflux surveyed in the treated plots was Rh, and CO_2_ efflux surveyed in the untrenched subplots was Rs. The difference between treated subplot and untrenched subplot was Ra. This root exclusion method has been widely used in the field to partition Ra and Rh^25,66,67^.

Soil CO_2_ flux was measured from May to October in 2014 and 2015 with a portable automated soil CO_2_ flux system (LI-8100 A, LI-COR Inc., Lincoln, USA). A PVC collars (20 cm diameter and 15 cm height) were inserted into each untrenched and trenched subplots to a depth of 3–5 cm below the surface for soil CO_2_ flux measurement one day before each survey day. The soil was tamped outside of the collar to prevent air leakage. All soil CO_2_ flux measurements were taken between 09:00 and 14:00 on sunny days. Before the measurements, all plants in each PVC collar were clipped. The measurements were conducted twice a month (early and late in the month) for four days each time. ST5 and SM10 were measured simultaneously near each PVC collar along with the Rs measurement by a probe connected to the LI-8100 A.

### Vegetation structure and soil properties

Vegetation and soil sampling were conducted in early September of 2014 and 2015. In each plot, eight 1 m × 1 m quadrats were randomly established 1 m from the edge. In each quadrat, the height and density of each plant and the aboveground, belowground and litter biomass were investigated. Aboveground biomass was cut at ground level for species and all litter in each quadrat was collected by hand. We used a 10 cm inner diameter auger to measure the root biomass in 0–10, 10–20 and 20–30 cm soil layer in each harvested quadrat. We collected eight soil cores at each plot, and twenty-four soil cores per N-addition treatment. We separated out the root by washing the soil samples within a 0.5-mm mesh bag. The plant, litter and root samples were first dried at 105 °C for 30 min, and then dried at 65 °C to constant weight. We used a cutting ring (100 cm^3^ volume) to measure the soil bulk density at 0–30 cm soil depth in each of the harvested quadrat (i.e., the soil was divided into 3 layers of 10 cm each).

We used a 3.5 cm inner diameter auger to take soil samples in each harvested quadrat (3 layers of 10 cm each) to measure SOC, TN contents. Eight cores were collected in each plot, and twenty-four soil cores per N-addition treatment. Soil samples were air-dried and sieved to pass a 2 mm mesh sieve for testing the SOC and TN. Meanwhile, the surface layer (0–10 cm) soil was collected with a 3.5 cm inner diameter auger to measure the SMBC content in each harvested quadrat. Eight cores were collected in each plot, and twenty-four soil cores per N-addition treatment. The soil samples were immediately sieved through a 2-mm screen in the field and kept refrigerated.

### Chemical analysis

The plant, litter, root and soil organic carbon contents were measured using a total organic carbon analyzer (Multi N/C 2100 s, Analytik Jena Germany). The plant, litter, root and soil total N contents were measured via the Kjeldahl method^68^. The SMBC content was measured via the chloroform fumigation extraction method. The carbon in the extract was measured through dichromate oxidation method, and the conversion coefficient was 0.38^69^.

### Statistical analysis

The plant C storage and N storage were calculated by the methods of Fang *et al*.^70^; the soil organic C storage and N storage were calculated using the methods of Ma *et al*.^71^. The C storage and N storage in the alpine meadow ecosystem were considered the sum of the plant, root, litter and soil C storage and N storage, respectively.

Repeated-measures ANOVA was applied to compare the significant differences in Rs, Ra and Rh, biotic and abiotic variables among the N-addition treatments. Considering the strong correlations among C and N storage in plant, litter, root, 0–10 cm soil layer, 10–20 cm soil layer, 20–30 cm soil layer, we used the PCA to determine the primary axes of covariation among the variables. Based on the PCA results, we chose the parameters with loading value greater than 0.900 and soil temperature and volumetric soil moisture as predictors to establish the SEM to identify the direct and indirect effect pathways on soil respiration. SEM was conducted with the Amos software program ver. 21.0.

Meanwhile, linear regression was conducted with the Origin software program ver. 8.5 to examine the relationships between soil respiration components (Rs, Ra and Rh) and the following: root C storage, 0–10 cm soil layer C storage, 10–20 cm soil layer C storage, soil temperature and soil moisture. The equation was as follow:

1 $${\rm{R}}={\rm{a}}+{\rm{bx}}$$

where R is Rs, Ra, or Rh; x is root C storage, 0–10 cm soil layer C storage, 10–20 cm soil layer C storage, soil temperature or soil moisture, and a and b are fitted parameters.

We used the following multiple linear regression to test the combined effect of root carbon storage, 0–10 cm layer carbon storage and 10–20 cm layer carbon storage on soil respiration rate (conducted with the Origin software program ver. 8.5):

2 $${\rm{R}}={\rm{c}}\times {\rm{RCS}}+{\rm{d}}\times {{\rm{SOCS}}}_{10}+{\rm{e}}\times {{\rm{SOCS}}}_{20}+{\rm{f}}$$

where R is Rs, Ra, or Rh, RCS is root carbon storage, SOCS_10_ is 0–10 cm layer carbon storage, SOCS_20_ is 10–20 cm layer carbon storage, and c, d, e and f are fitted parameters.

We used the following multiple linear regression to test the combined effect of soil temperature and moisture on soil respiration rate (conducted with the Origin software program ver. 8.5):

3 $${\rm{R}}={\rm{g}}\times {\rm{ST}}5+{\rm{h}}\times {\rm{SM}}10+{\rm{i}}$$

where R is Rs, Ra, or Rh, ST5 is soil temperature at 5 cm depth, SM10 is volumetric soil moisture at 10 cm depth, and g, h, and i are fitted parameters.

All statistical analyses were conducted using the SPSS software program ver. 19.0 (SPSS, Chicago, IL).
